# Aphid colony duration does not limit the abundance of *Harmonia axyridis* in the mediterranean area

**DOI:** 10.1038/s41598-020-78257-7

**Published:** 2020-12-03

**Authors:** Pavel Kindlmann, Zuzana Štípková, Anthony F. G. Dixon

**Affiliations:** 1 Department of Biodiversity Research, Global Change Research Institute, Bělidla 986/4a, 60300 Brno, Czech Republic; 2 Institute for Environmental Studies, Faculty of Science, Charles University, Prague, Czech Republic; 3 School of Biological Sciences, University of East Anglia, Norwich, UK

**Keywords:** Ecology, Zoology

## Abstract

There is a lot of information on the factors limiting the distributions of species in their native areas, but much less on those limiting potential changes in distributions of species that are currently spreading outside their present range, especially invasive species. However, this information is often quite essential, as it enables the prediction of future spatial distributions and local abundances of invasive species and the potential effect they may have on people and crops. Arising from glasshouse escapes in North America and the Netherlands, the invasive ladybird, *Harmonia axyridis* (Pallas) (Coleoptera: Coccinellidae), originally from the east Palearctic, has now spread across the whole of North America and most of Europe, both of which caused serious concern. Recent observations show that the spread of *H. axyridis* towards the equator is limited. For example, it is quite rare in the Mediterranean area and its northward spread in South America is also quite slow, as if there was something limiting its spread towards the equator. European literature indicates it is neither climate, nor the distance of the Mediterranean from the original release location in the Netherlands. Therefore, we hypothesized that it may be biotic factors. In particular, the duration of colonies of prey (colony is the set of individuals in one patch, usually on one plant) that becomes shorter as one approaches the equator. This may lower the fitness of the predator and subsequently lead to low population densities. We test here, whether the duration of aphid colonies is shorter in the Mediterranean area than in Central Europe. Unfortunately, our data does not support this hypothesis. Therefore, the question of what limits the distribution of *H. axyridis* towards the equator remains to be resolved.

## Introduction

A species’ distribution is determined by three main factors: (1) original location, where the species evolved, (2) population ability to spread outside the original location and (3) ecological and environmental factors limiting its spread^1^. Of the ecological and environmental factors, climate, particularly temperature, is often assumed to limit the occurrence of a species in a particular region. Several models predicting the distributions of species using meteorological data (CLIMEX^2,3^, SDM models^4^) are used to predict the survival probability of a species outside its native distribution^2^ or the extent of the area that may be potentially occupied by a species^4,5^.

There is a lot of information on the factors limiting the distributions of species in their native areas^1^, but much less information on the factors limiting potential changes in distributions of species that are currently spreading outside their present range, especially that of invasive species^6^. This information is often quite essential, as it enables the prediction of future spatial distributions and local abundances of invasive species and their potential effect on people and crops. Information on potential spread of a species also helps with the prediction of the effect of climate change on (1) changes in species distributions, (2) likelihood of their extinction associated with these changes and (3) subsequent threat to humans or crops^7^.

Ladybirds are an ideal model group for testing the limiting effects of various factors on changes in the distributions of invasive species. This is because: (1) coccinellid biology is well studied, as they are potentially good biological control agents^8–13^, (2) they are conspicuous and mostly highly abundant and (3) invasive species are frequently reported in this group^14^ either as a consequence of intentional (ladybirds have been successfully introduced to new geographic regions since the early 1900s) or unintentional introductions^14^.

We use the invasive ladybird, *Harmonia axyridis* (Pallas), as a model species, mainly because its spread in North America and most of Europe is a matter of serious concern^15^. The original area of its distribution is east Palaearctic (Japan, Korea, China, southern Siberia). The early attempts to introduce this species, a voracious predator of aphids and some other insect pests, from its native area into areas with a climate suitable for its survival, were not successful^16^. Later this species was mass-produced and used as an efficient means of biological control in glasshouses, mostly against aphids. Some of these domesticated populations escaped from glasshouses and spread into the surrounding environment, first in North America^17^. Later, *H. axyridis* started to spread into areas with mild-climates around the world: first in Europe after escaping from glasshouses in the Netherlands, then in South America and also in tropical areas^15^.

In mainland Europe, there is considerable latitudinal variation in the abundance of *H. axyridis*^18^, which dominate mainland communities’ north of 46° N, while it is rare in those that are south of 42° N. Also, according to unpublished preliminary data^14,18^, *H. axyridis* is almost absent in the Mediterranean area. The question therefore arises, why the spread of *H. axyridis* towards the equator is limited and whether or not this is a general phenomenon that also holds for other species. Is it because of climatic factors, or the large distance of the Mediterranean from the initial release point in the Netherlands, or is it something else?

Both SDM and CLIMEX models, based on climatic data, predict that the Mediterranean region is suitable for *H. axyridis* and therefore one would expect it to be abundant there^3,4^, so it does not seem to be climate that limits the abundance of *H. axyridis* in warmer regions. This idea is further supported by the studies made in South America. Grez et al.^19^ report a rapid spread of *H. axyridis* south and a slow spread north of 33° S, where it was initially introduced near Santiago de Chile. Again, the models of spread of *H. axyridis* based on climatic data^5^ predict exactly the opposite. Thus, assuming that the SDMs’ predictions are correct, it is unlikely that the factor that limits the geographic distribution of this species towards the equator can be climate. Neither is the rarity of *H. axyridis* in the Mediterranean area likely to be due to a slow rate of its southward dispersal from a distant source area in Western Europe, as its observed rate of spread in a west–east direction is more than 300 km/year^15^.

Therefore, we hypothesized that the reason might be due to biotic factors. Here we focus on the most likely: diet and life histories. We hypothesized that the limiting factor can be the duration of colonies of prey (i.e., number in one patch, usually on one plant) that may shorten as one approaches the equator. It is well known that there exists a sharp change from mild Central European climate with long vegetative season to harsh Mediterranean conditions with a short vegetative season, which is reflected in the composition of animal communities^20,21^, one result of which may be shortening of the duration of aphid colonies. As indicated by mathematical models of ladybird—aphid systems, short duration of the prey (aphid) colony may lead to a lower fitness and subsequently low population densities of the predator^22–25^. Thus, abundance of this predator is most likely to be positively correlated with the duration of the prey colonies necessary for the completion of its larval development.

To summarize: we test whether the duration of aphid colonies is shorter in the Mediterranean area than in Central Europe and whether this limits the abundance of *H. axyridis* in the Mediterranean area.

## Results

The species of aphids monitored in the field, when possible, were determined along with their host plants. We were not able to determine all of them for Greece as some may be new for this country. Scientific names of aphids and their host plants are listed in Table 1.

**Table 1:** Aphids found on plants during the field studies in Greece (in 2017 and 2018) and in the Czech Republic (in 2019).

Year	Country	Host plant	Aphid species
2017	Greece	*Malus domestica*	*Dysaphis plantaginea*
		*Brassica oleracea*	*Brevicoryne brassicae*
		*Prunus persica* *Prunus cerasifera*	*Hyalopterus pruni*
		*Rubus idaeus*	*Aphis idaei*
		*Rumex cristatus*	*Aphis fabae, Aphis* sp.
		*Sonchus asper*	*Uromelan sonchi*
2018	Greece	*Brassica oleracea*	*Brevicoryne brassicae*
		*Hieracium laevigatum*	*Aphis hieracii*
		*Hieracium pilosella*	*Urolecon pilosellae*
		*Rubus idaeus*	*Amphorophora rubi, Aphis idaei*
		*Rumex cristatus*	*Aphis* sp.
		*Sonchus asper*	*Uromelan sonchi*
2019	Czech Republic	*Artemisia vulgaris*	*Macrosiphoniella artemisiae*
		*Matricaria chamomilla*	*Aphis* sp.
		*Cirsium arvense*	*Aphis fabae, Aphis* sp.
		*Gallium aparine*	*Aphis* sp.
		*Leontodon hispidus*	*Uroleucon leontodontis*
		*Rumex obtusifolius*	*Aphis fabae, Aphis* sp.

**Figure 1:**
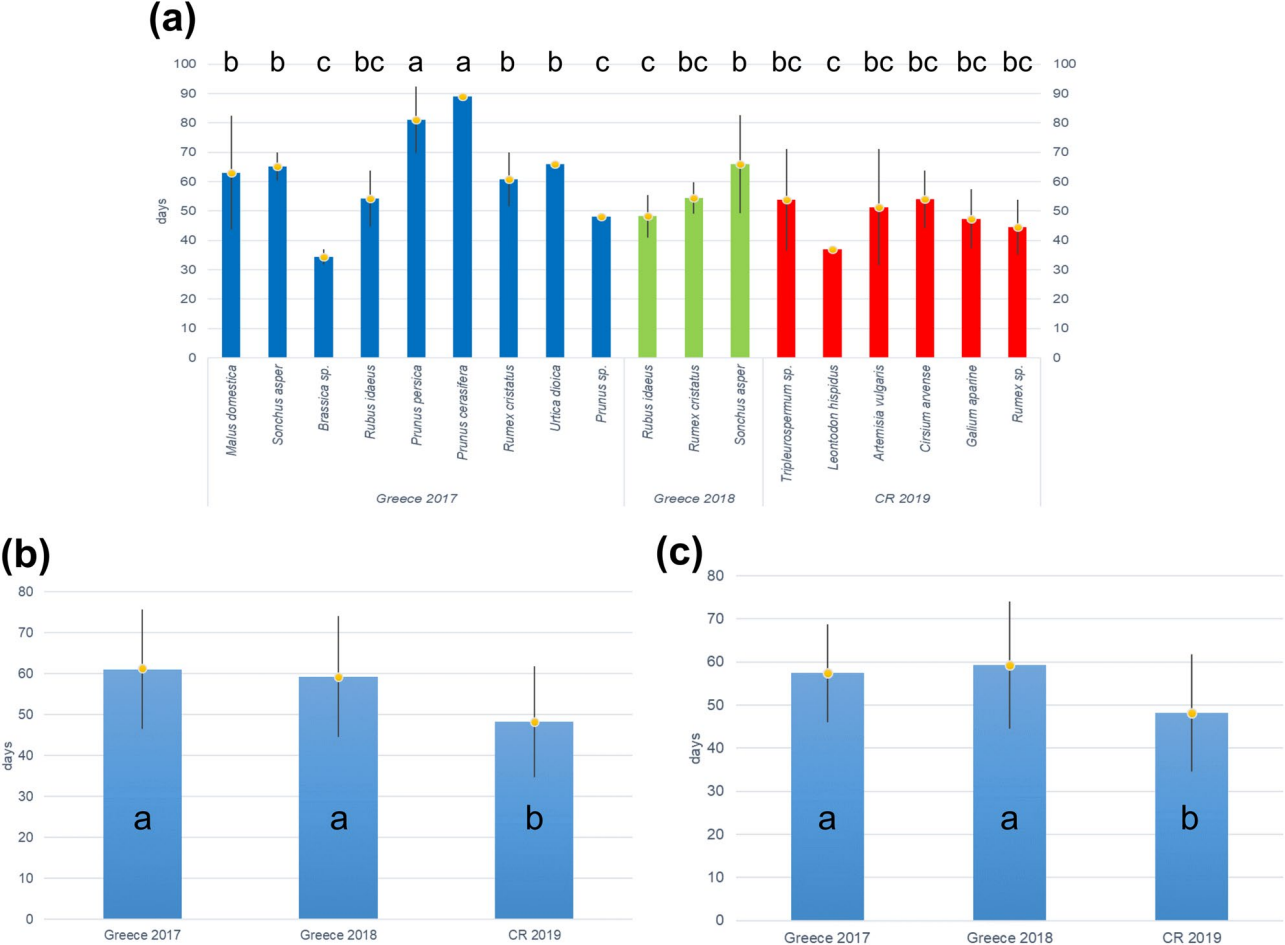
Average aphid colony duration in days: (**a**) on individual species of plants; (**b**) average for one year and country for all plants monitored (including trees) (**c**) average for one year and country only for herbaceous plants. Different letters above the columns in (**a**) and inside the columns in (**b**) and (**c**) indicate significantly different values (Tukey HSD test). Vertical bars indicate SD.

Figure 1 shows the duration (in days) of aphid colonies in both countries and years. The numbers displayed in Fig. 1a are the averages for aphid colony duration on individual species of plants. We also analysed the raw numbers in addition to the averages and the results were very similar, so only average numbers are presented. Single factor ANOVA revealed significant differences between the three datasets (F = 5.30, p < 0.05). The subsequent Tukey HSD test revealed that colony duration in the Czech Republic in 2019 on herbaceous plants (48 days) was significantly shorter than in Greece both in 2017 and 2018, while there was no significant difference in colony duration in 2017 and 2018 in Greece (60 and 59 days, respectively).

Figure 1b shows similar results to Fig. 1a, but with data for individual species within one year and one country pooled. Figure 1c shows the same as Fig. 1b, but with data for trees excluded. Not surprisingly, in both Fig. 1b,c, single factor ANOVA also revealed significant differences between the three datasets (F = 12.74, p < 0.05; F = 9.27, p < 0.05) and the subsequent Tukey HSD test revealed that colony duration in the Czech Republic in 2019 was significantly shorter than in Greece in both 2017 and 2018, while there was no significant difference between colony duration in 2017 and 2018 in Greece.

**Figure 2:**
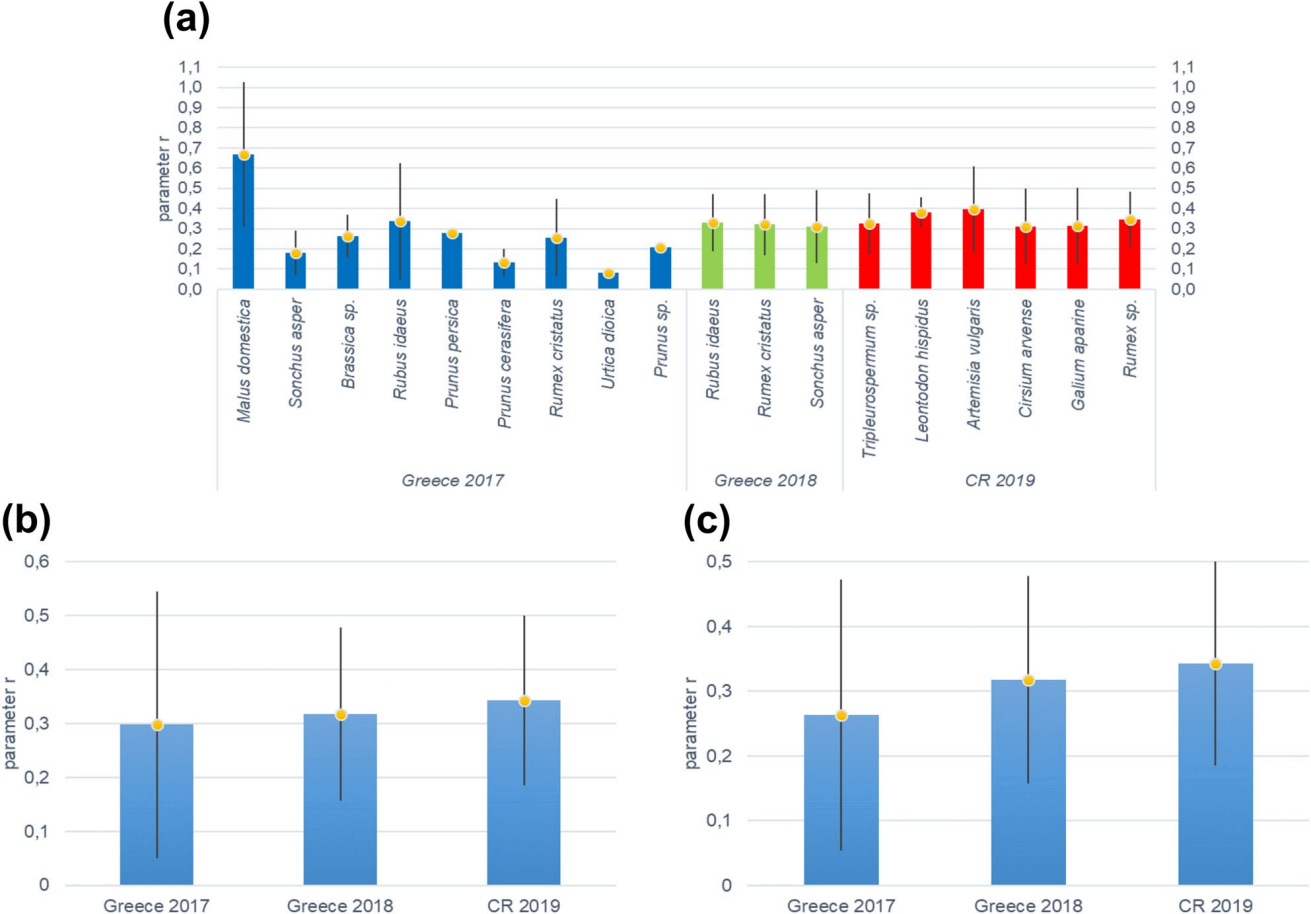
Average aphid population growth rate, *r* for colonies: (**a**) on individual species of plants; (**b**) average for one year and country for all plants monitored (including trees) (**c**) average for 1 year and country only for herbaceous plants.

Figure 2a shows the average values for the instantaneous growth rate of the aphid populations (parameter *r*) on individual species of plants. Single factor ANOVA did not reveal significant differences between the values of *r* (F = 1.49, p > 0.05). Figure 2b shows the overall average values of parameter *r* across the whole season in both countries when trees were sampled. Single factor ANOVA did not reveal significant differences between the values of *r* (F = 0.70, p > 0.05). The lowest average population growth rate of aphids was recorded in Greece in 2017 and the highest in the Czech Republic in 2019, but none of the differences between these averages were statistically significant (the ANOVA above). Figure 2c shows the overall average values of parameter *r* in both countries when trees were excluded from the analysis. The growth rate of aphid colonies in Greece in 2017 was slightly lower, but the results of the statistical analyses remained the same as in Fig. 1b (F = 2.22, p > 0.05). In general, the results tend to show no significant difference in the growth rates.

**Figure 3:**
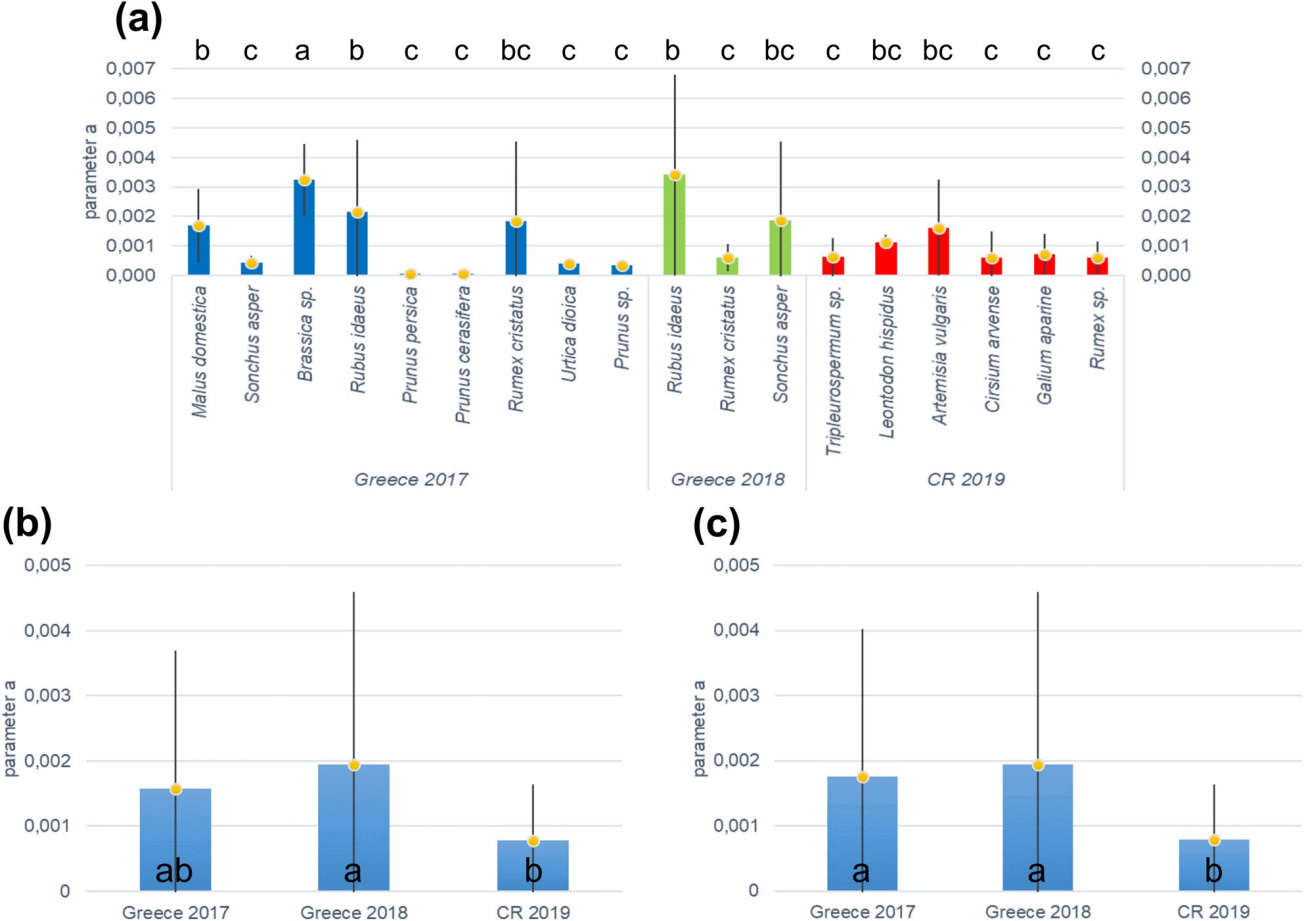
Average values of parameter *a* in Eq. (1) for colonies: (**a**) on individual species of plants; (**b**) average for one year and country for all plants monitored (including trees) (**c**) average for one year and country only for herbaceous plants. Different letters above the columns in (**a**) and inside the columns in (**b**) and (**c**) indicate significantly different values (Tukey HSD test). Vertical bars indicate SD.

Figure 3a shows the average values of the intensity of regulation (parameter *a*) on individual species of plants. It is the equivalent of carrying capacity used in logistic equations. The average values of parameter *a* fluctuated greatly both between individual species of plants, and between countries and years. Single factor ANOVA revealed significant differences between the values of *a* (F = 1.81, p < 0.05). It is not clear, why these values were high for some species and low for others. Single factor ANOVA also revealed significant differences between the values of *a* in Fig. 3b,c (F = 4.80, p < 0.05; F = 5.24, p < 0.05).

## Discussion

The duration of an aphid colony on a plant is very likely to affect the number of ladybirds that complete their development. This is predicted by theoretical models^11,13,23,25^ and is also recorded in field data, as the incidence of cannibalism and death due to starvation increases during the fourth instar, i.e., towards the end of the existence of an aphid colony^9,26^. Thus, when looking for a reason for the paucity of *H. axyridis* in the Mediterranean, we considered duration of aphid colonies as one possible candidate.

It might be argued that the hypothesis that differences in colony duration cause the paucity of *H. axyridis* in the Mediterranean is irrelevant, because a considerable percentage of ladybirds complete their whole development on trees, where aphids, if they are not host-alternating, are present during the whole season. Considering this argument, two cases may occur: (1) if most ladybirds develop on trees that host non-host alternating aphid species, then colony duration cannot be responsible for lack of *H. axyridis* in the Mediterranean area, because these aphids occur on such trees throughout the vegetative season and so can the ladybirds. Thus, colony duration has no effect in this case. (2) If a considerable number of ladybirds develop on herbaceous plants, then our findings (longer colony duration in Greece than in the Czech Republic) also show that colony duration cannot be responsible for lack of *H. axyridis* in the Mediterranean area.

It is also noteworthy that the counterintuitive trend in aphid colony duration (longer colony duration in the Mediterranean area) was also not caused by other factors. No trends were found in the values of parameters *r* and *a* between years and countries, which means that these values could not affect colony duration.

Choice of plants was haphazard and we monitored all aphid colonies with less than ten individuals found along the transects. One could argue that because of the large numbers of species of host plants and their different abundancies the results were biased. In theory, this might be possible, but the results were statistically significant and the number of plants monitored was the maximum that could realistically be sampled.

**Figure 4:**
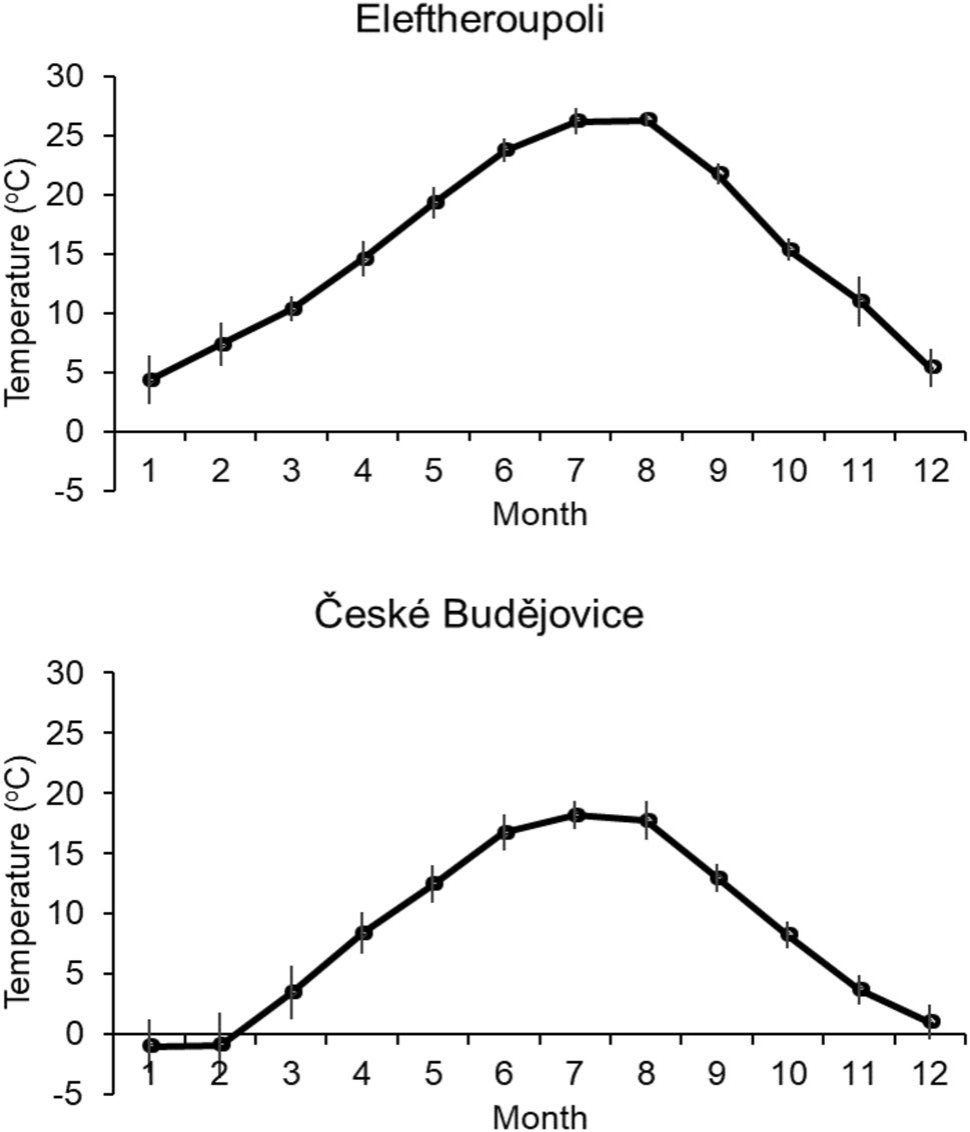
Ten-year averages (+ SDs) of the average monthly temperatures for Eleftheroupoli in Greece, which is close to Néa Péramos, and for České Budějovice, which is one of the Czech sites.

Another criticism may stem from the fact that the data for the different countries were collected in different years, but even this factor does not seem to have affected the outcome for two reasons:

*First* even if the weather in the Czech Republic in 2019 was warmer than usual, it was still much colder there than in the Mediterranean area (see Fig. 4).

*Second* in principle, we have one response variable—colony duration, which may be influenced by two factors: site location (temperate region vs Mediterranean) and the year when the data was collected. We are testing the following hypothesis.

### H0

Colony duration is significantly shorter in the Mediterranean, independently of the year of monitoring.

Based on the data collected, there can be two conclusions:

(A) Colony duration *is* significantly shorter in the Mediterranean. However, if the data were collected in different years, then it is not possible to determine whether the observed shorter colony duration in the Mediterranean was caused by different sites (Mediterranean vs. temperate region), or by different years of monitoring and further years of monitoring are required, before the claim that H0 is supported by the data is substantiated. This, however, is not our case.
(B) Colony duration *is not* significantly shorter in the Mediterranean (there is either no difference, or the opposite holds—*the latter is our case*). Then again there are two options:
  1. *There is no effect of year.* In such a case, we should *reject H0*. In our data, the non-significant effect of year in the Greek data *supports*, but does not *prove* no effect of year. The latter is because: (1) we compared only two years and (2) the same test was not performed in the temperate region, for which there is only one year of data.
  2. We suspect *there might be an effect of year* (and based on our data we cannot exclude this with certainty—see above). Then, if the data were collected in different years (as in our case), our conclusion must be weaker than rejection, hence *our data do not support H0.*

As explained above, based on (B1), the conclusion cannot be stronger. Thus, to be safe, we stay with the conclusion based on (B2). This conclusion is not a statement of the obvious. Longer colony duration in the Mediterranean (although based on data from different years) is an unexpected result, based on a large amount of data. So our data *do not support the idea that shorter colony duration is the reason for the paucity of Harmonia in the Mediterranean*, as colony duration was not *shorter* there.

One could also criticize the low number of replicates in terms of sites and years. In terms of effort, one or two people spent 10 months of uninterrupted day-to-day monitoring in the field (five in each country). The results are statistically significant and indicate the opposite to what was expected. Of course, it would be better to have had three sites in each country and monitored each of the colonies at each site for 3 years. This would require not 10, but 23 months of interrupted day-to-day monitoring by 1–2 people. In the future, funding may become available to do this, but meantime, accepting the statistically significant results and looking for other possible reasons for the paucity of *H. axyridis* in the Mediterranean would appear to be the better strategy.

## Materials and methods

**Figure 5:**
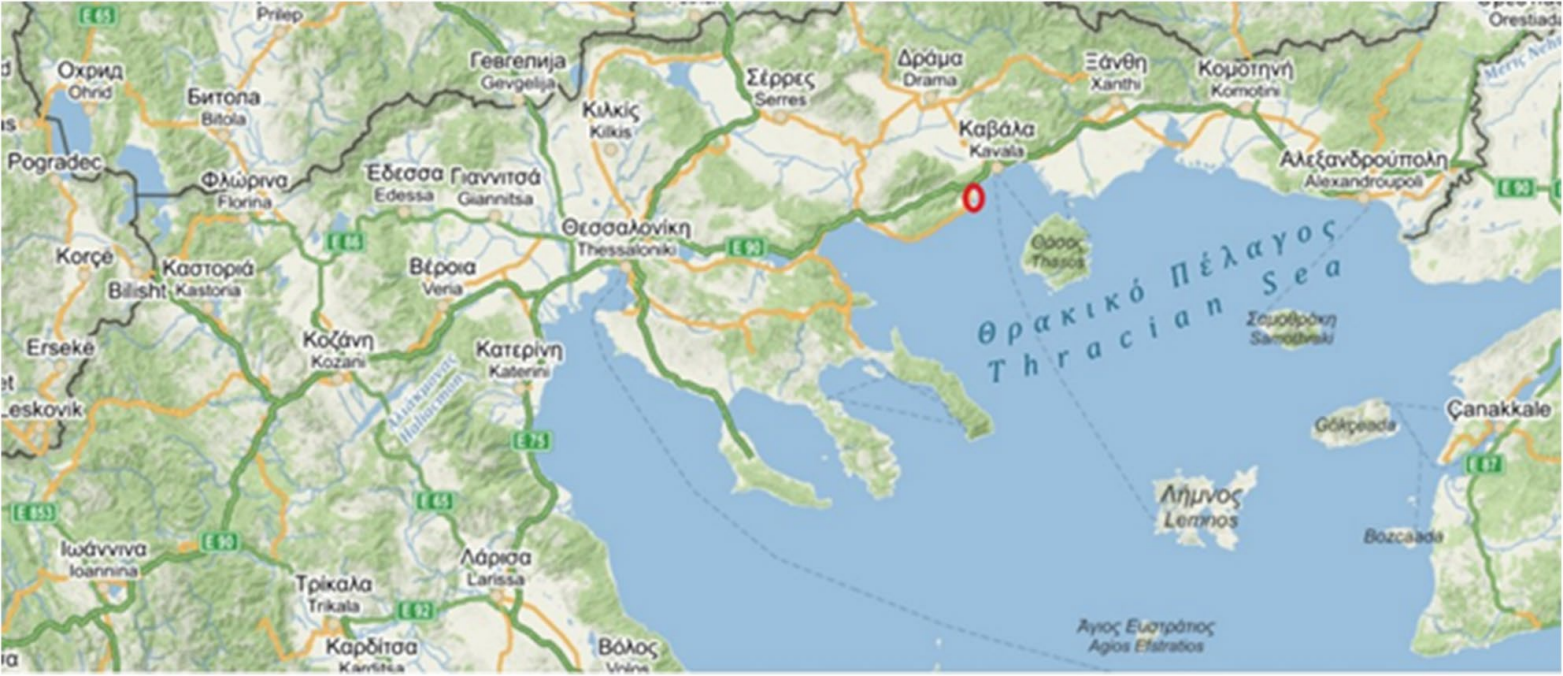
Map of the area sampled in Greece (Mediterranean). The red circle shows the location of Néa Péramos (modified from the open source www.mapy.cz).

**Figure 6:**
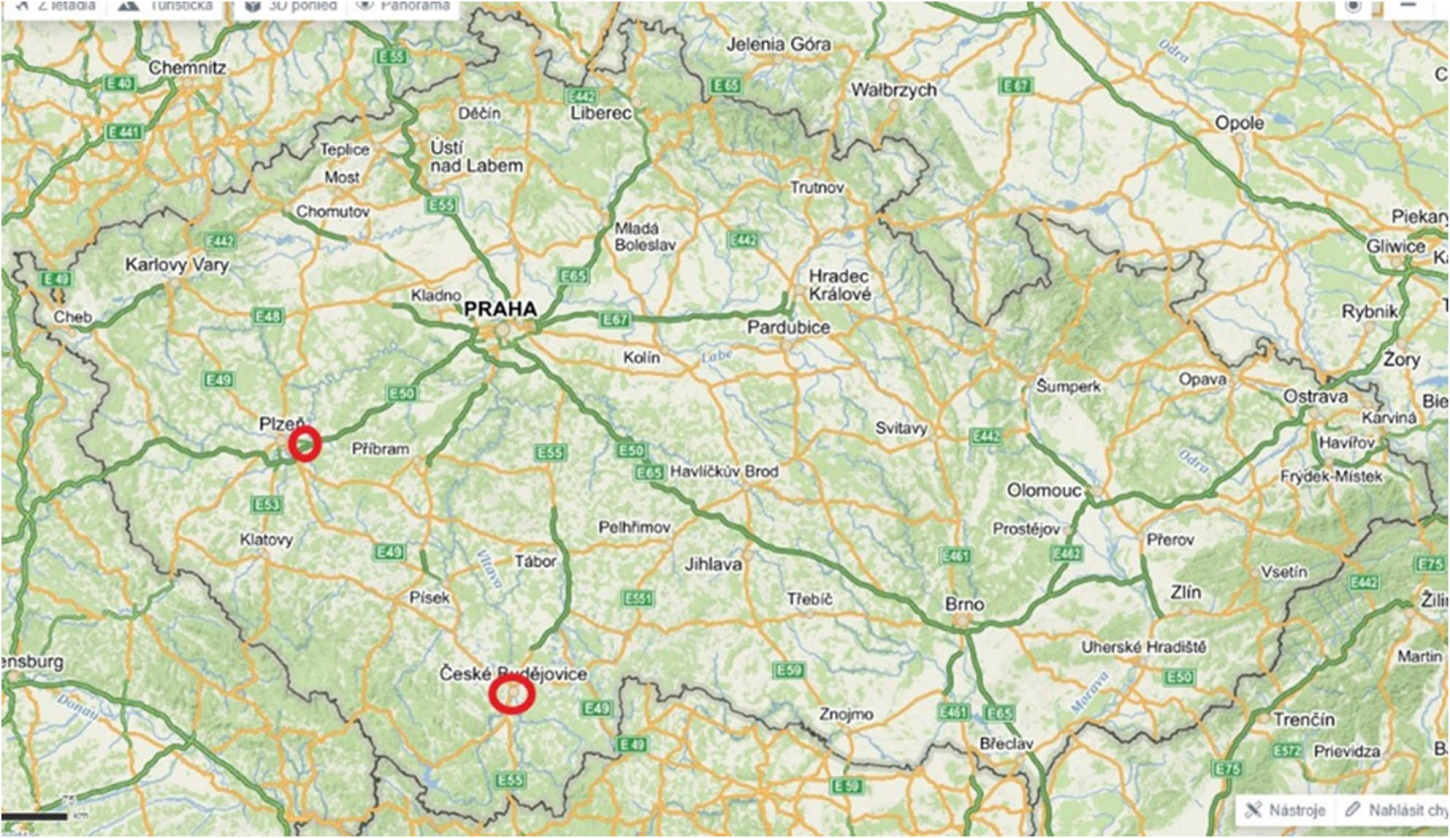
Map showing the locations of the two sites sampled in the Czech Republic, near České Budějovice and close to Kyšice near Plzeň (modified from the open source www.mapy.cz).

The data for the Mediterranean area were collected in north-eastern Greece around the village Néa Péramos, which is situated on the coast (Fig. 5). We spent a total of five months there during 2017 and 2018, from April to mid-June each year, which is when the aphid colonies there are present. The data for the Czech Republic was collected at two places, around Kyšice village near Plzeň and in the surroundings of České Budějovice in 2019, from the end of April to the beginning of August (Fig. 6). The two different datasets for the Czech Republic were pooled prior to the analyses, because t-test revealed that there is no significant difference between colony durations at these two sites (t = − 1.355, P = 0.18) and it resulted in a larger and compact dataset for the Czech Republic.

Colony duration is expected to be shorter in Greece than in the Czech Republic, because the climate in Greece is warmer than in the Czech Republic. This is commonly known, but is further confirmed by Fig. 4, where 10-year averages (± SDs) of monthly temperatures are plotted for Eleftheroupoli in Greece, which is close to Néa Péramos (as a representative of the Mediterranean climate see, e.g., https://en.wikipedia.org/wiki/Mediterranean_climate), and for České Budějovice, which is one of the Czech sites. The average monthly temperatures for the other Czech site, Plzeň are almost exactly the same as those for České Budějovice and therefore are not presented here.

We looked for aphid colonies on all plants along haphazardly located 3–5 km transects around the villages, but only monitored those on herbaceous plants and of host-alternating species on trees. The host specific aphids on trees were not included in this study as they are often present throughout the vegetative season. These aphids are also attacked by natural enemies at times when they are actively reproducing^27^, but as these aphids are present throughout the vegetative season, it is difficult to determine the duration of the period when they are likely to be attacked by natural enemies.

In order to measure colony duration only those colonies were monitored that were in a very early stage in their development (this means that only up to ten aphids were present on a plant and there were no remnants of dead aphids). For all the colonies monitored, their GPS coordinates were recorded and a colour label with a specific code was attached to the plant. These coordinates only served to distinguish individual colonies and are not reported here. From the length of the transects it follows that the colonies were very close to each other and their position in space is clear from the position of the city.

In every aphid colony monitored, the numbers of all the aphids present were recorded. For monitoring aphid population dynamics, we visited marked colonies every 3 days and counted the aphids and their enemies on the whole plant.

To describe aphid population dynamics, data for each colony were fitted by the aphid model

1 $$x^{\prime}\left( t \right) = rx\left( t \right)\left[ {1 - a\int\limits_{0}^{t} {x\left( \tau \right)d\tau } } \right] + i,$$

where *x*(*t*) is the number of aphids at time *t* and *r* (instantaneous growth rate of the aphid population), *a* (intensity of regulation, analogous to carrying capacity used in logistic equations) and *i* (negative *i* indicates emigration from the colony, positive *i* means immigration from outside) are parameters. In this model, instead of carrying capacity, used in the logistic equation, we used aphid cumulative density (the cumulative number of aphids from the beginning of colony existence) scaled by the parameter *a*^28^.

For estimating these parameters, the Euler method^29^ was used. This model gave us an unbiased estimate of the end of the existence of an aphid colony. Of course, we also recorded the real end of the colony, but this date was affected by many random factors. So, we preferred to extrapolate forwards based on the data on the colony population dynamics and on the model predictions. The instant, when the model predicted a colony size equal to 1, was assumed to be the end of the colony.

To get an estimate of the beginning of colony development, we extrapolated the number of aphids backwards in time using an exponential function, the exponent of which was fitted based on the subsequent dynamics, until we reached a predicted colony size 1. As all the colonies monitored were chosen based on being very close to the beginning of their development when first recorded, this backward extrapolation was quite a precise method of estimating the actual beginning of the development of a colony.

After careful checking whether all the assumptions of the tests used were satisfied, we used a single factor ANOVA and a subsequent Tukey HSD test (if ANOVA indicated presence of significant differences) were used to test for differences between aphid colony duration using the three datasets. We performed these analyses not only for data on the duration of aphid colonies, but also for the aphid population growth rate, *r*, and the parameter *a* in Eq. (1). This was because we were interested in whether the differences between these values (if present) may have affected colony duration: e.g., difference in *r* might have speeded up the growth of aphid numbers in the colony, which in turn might have affected colony duration.

The monitoring of colonies of only host alternating species of aphids on trees might have biased the result because trees are much larger than herbaceous plants and therefore the duration of the colonies might be longer on trees than on herbaceous plants. Of the haphazardly selected plants, the duration of colonies of aphids on trees were only recorded in Greece in 2017. Therefore, to address this possible bias, the analyses mentioned was carried out twice: once including trees and once without, to determine whether including aphid colonies on trees biased the result.

## Conclusions

We hypothesized that aphid colony duration in Greece is shorter as the climate there is warmer. Our empirical data indicate the opposite: duration of aphid colonies in Greece was significantly longer than in the CR. This does not support our hypothesis. Therefore, colony duration does not seem to be the reason for the low numbers of *H. axyridis* in Greece. In the future, there is a need for more extensive studies, including more years, more countries, more species of plants per year and country, to deal with the potential biases in this study. It is a pity that limitations on funding prevented the simultaneous monitoring in both countries in the same years and for more years. In the absence of this constraint, our results might have been more robust. However, even the results presented here indicate that more extensive studies are unlikely to add anything new, as the paucity of *H. axyridis* in the Mediterranean area is most probably due to factors not yet considered.

## Data Availability

All datasets generated and/or analysed during the current study are available in the Global Change Research Institute CAS, Prokišova 356/7, 37001 České Budějovice, Czech Republic. We can provide the data to individual researchers upon formal request to the authors.
